# Residual Effect of Texting to Promote Medication Adherence for Villagers with Schizophrenia in China: 18-Month Follow-up Survey After the Randomized Controlled Trial Discontinuation

**DOI:** 10.2196/33628

**Published:** 2022-04-19

**Authors:** Yiyuan Cai, Wenjie Gong, Wenjun He, Hua He, James P Hughes, Jane Simoni, Shuiyuan Xiao, Stephen Gloyd, Meijuan Lin, Xinlei Deng, Zichao Liang, Bofeng Dai, Jing Liao, Yuantao Hao, Dong Roman Xu

**Affiliations:** 1 Department of Epidemiology and Health Statistic School of Public Health Guizhou Medical University Guiyang China; 2 HER Team and Department of Maternal and Child Health Xiangya School of Public Health Central South University Changsha China; 3 Department of Psychiatry University of Rochester Medical Center Rochester, NY United States; 4 Institute of Applied Health Research University of Birmingham Birmingham United Kingdom; 5 Center for World Health Organization (WHO) Studies and Department of Health Management School of Health Management of Southern Medical University Guangzhou China; 6 Acacia Labs, Institute for Global Health and Dermatology Hospital Southern Medical University Guangzhou China; 7 School of Public Health and Tropical Medicine Tulane University New Orleans, LA United States; 8 Department of Biostatistics University of Washington Seattle, WA United States; 9 Department of Psychology University of Washington Seattle, WA United States; 10 Department of Global Health University of Washington Seattle, WA United States; 11 Xiangya School of Public Health Central South University Changsha China; 12 School of Nursing The Hong Kong Polytechnic University Hung Hom, Kowloon, Hong Kong Special Administrative Region China; 13 School of Public Health Sun Yat-sen University Guangzhou China

**Keywords:** medication adherence, mobile texting, lay health worker, resource-poor community, primary health care, quality of care, mHealth, schizophrenia, maintenance, residual effect, mental health, patient outcomes

## Abstract

**Background:**

Reducing the treatment gap for mental health in low- and middle-income countries is a high priority. Even with treatment, adherence to antipsychotics is rather low. Our integrated intervention package significantly improved medication adherence within 6 months for villagers with schizophrenia in resource-poor communities in rural China. However, considering the resource constraint, we need to test whether the effect of those behavior-shaping interventions may be maintained even after the suspension of the intervention.

**Objective:**

The aim of this study is to explore the primary outcome of adherence and other outcomes at an 18-month follow-up after the intervention had been suspended.

**Methods:**

In a 6-month randomized trial, 277 villagers with schizophrenia were randomized to receive either a government community mental health program (686 Program) or the 686 Program plus Lay health supporters, e-platform, award, and integration (LEAN), which included health supporters for medication or care supervision, e-platform access for sending mobile SMS text messaging reminders and education message, a token gift for positive behavior changes (eg, continuing taking medicine), and integrating the e-platform with the existing 686 Program. After the 6-month intervention, both groups received only the 686 Program for 18 months (phase 2). Outcomes at both phases included antipsychotic medication adherence, functioning, symptoms, number of rehospitalization, suicide, and violent behaviors. The adherence and functioning were assessed at the home visit by trained assessors. We calculated the adherence in the past 30 days by counting the percentage of dosages taken from November to December 2018 by unannounced home-based pill counts. The functioning was assessed using the World Health Organization Disability Assessment Schedule 2.0. The symptoms were evaluated using the Clinical Global Impression–Schizophrenia during their visits to the 686 Program psychiatrists. Other outcomes were routinely collected in the 686 Program system. We used intention-to-treat analysis, and missing data were dealt with using multiple imputation. The generalized estimating equation model was used to assess program effects on adherence, functioning, and symptoms.

**Results:**

In phase 1, antipsychotic adherence and rehospitalization incidence improved significantly. However, in phase 2, the difference of the mean of antipsychotic adherence (adjusted mean difference 0.05, 95% CI −0.06 to 0.16; *P*=.41; Cohen *d* effect size=0.11) and rehospitalization incidence (relative risk 0.65, 95% CI 0.32-1.33; *P*=.24; number needed to treat 21.83, 95% CI 8.30-34.69) was no longer statistically significant, and there was no improvement in other outcomes in either phase (*P*≥.05).

**Conclusions:**

The simple community-based LEAN intervention could not continually improve adherence and reduce the rehospitalization of people with schizophrenia. Our study inclined to suggest that prompts for medication may be necessary to maintain medication adherence for people with schizophrenia, although we cannot definitively exclude other alternative interpretations.

## Introduction

### Background

Treatment with antipsychotic medication effectively prevents relapse and rehospitalization in people with schizophrenia [1-4]. However, 69% of people with schizophrenia in low- and middle-income countries have no access to any evidence-based care, often because of resource constraints or health system failures [5]. Even when treatment is available, adherence to antipsychotics is rather low [6], with nearly half of people with schizophrenia taking less than 70% of their prescribed doses [7]. In 2005, China launched the National Continuing Management and Intervention Program for Psychoses, also known as the 686 Program [8,9], which later became part of China's *integrated public mental health service*. The program followed the World Health Organization Mental Health Gap Action Program recommendations [10] and featured a collaborative approach between psychiatrists and community health workers to screen, diagnose, treat, and manage psychosis in communities [11,12]. In 2017, the 686 Program covered over 5,810,000 people with psychosis across China [11]. However, although the program provides free medication to the poor, over 70% of program enrollees failed to take their antipsychotic medications routinely [9].

China’s 686 Program, which is likely the largest community-based mental health program in the world, benefited a vast population of people with schizophrenia. However, the program effect had been reduced because of the poor patient adherence to antipsychotics [6,9]. Schizophrenia can often be effectively controlled with lifelong antipsychotic medications [4]. Mobile texting or SMS text messaging was found to be an inexpensive, easy-to-use, and reliable means to improve treatment adherence and schizophrenia care in some high-income settings [13-15]. To test its effectiveness in the resource-poor setting, we conducted a pragmatic trial featuring SMS text messaging reminders as the core of intervention in 2016 in 9 rural communities of China (called the Lay health supporters, e-platform, award, and integration [LEAN] Trial) [6,16]. The 6-month trial showed that LEAN significantly improved medication adherence (proportion of dosages taken) from 0.48 to 0.61 (adjusted mean difference 0.11, 95% CI 0.03-0.20; *P*=.007) among villagers with schizophrenia, and the incidence of rehospitalization due to schizophrenia decreased from 19.5% (25/128) under the control arm to 7.3% (9/124) under the intervention arm (relative risk 0.32, 95% CI 0.18-0.76; *P*=.007). However, other outcomes, including patient functioning and symptoms measured in phase 1, did not present significant improvement.

### Objectives

As of August 1, 2021, we have not yet identified other studies that have been explored the maintenance of the effect after a texting-based intervention was withdrawn in people with schizophrenia. Although we identified no similar studies that focused on the same participants as ours, studies of behavioral interventions using mobile texting in other populations suggested that the program effects lasted 8 to 12 weeks after the termination of texting [17]. Dobson et al [18] found that the impact of a texting intervention to improve self-management in patients with diabetes remained effective 15 months after the intervention was discontinued. Menon et al [19] found that the medication adherence in patients with bipolar I disorder persisted for at least three months after the intervention was discontinued. It is essential to understand the sustained effect of SMS text messaging after the intervention because (1) most interventions will not or cannot go on forever because of resource constraints or other reasons and (2) it may not be necessary to perpetuate a behavior-oriented intervention, as in theory, once people with schizophrenia habituate to new behavior, the original *reminder* may no longer be needed to stimulate the desired behavior. In the LEAN study, rural people with schizophrenia were randomized into either a mobile texting group (the LEAN intervention group) or a care-as-usual group (the control group) and were followed up for 6 months on LEAN’s effect on medication adherence, symptoms, and functioning. The LEAN intervention was then suspended, and we re-examined its sustained effect 18 months later. The study was divided into 3 phases. Phase 1 was a 2-arm randomized controlled study to evaluate the impact of LEAN in improving medication adherence. Phase 2 suspended the implementation of LEAN for the intervention group. Phase 3 restarted LEAN and applied it to both the original intervention and control groups and used information from all phases to evaluate the long-term effect of LEAN. Phase 1 and phase 3 results were published elsewhere [6,16], which established and reinforced the conclusion about LEAN’s impact on improving medication adherence. This paper reported the results of phase 2. The suspended implementation of LEAN during this phase provided a unique opportunity to understand whether texting can establish a new medication-taking behavior that may make texting no longer necessary. We hypothesized that the effects would be sustained as people may have habituated to the new behavior in the initial 6-month intervention so that the reminders may no longer be necessary. Furthermore, we also hypothesize that, with the improvement of the symptoms, functioning, and other outcomes would also present improvements in the long term.

## Methods

### Trial Design

**Figure 1:**
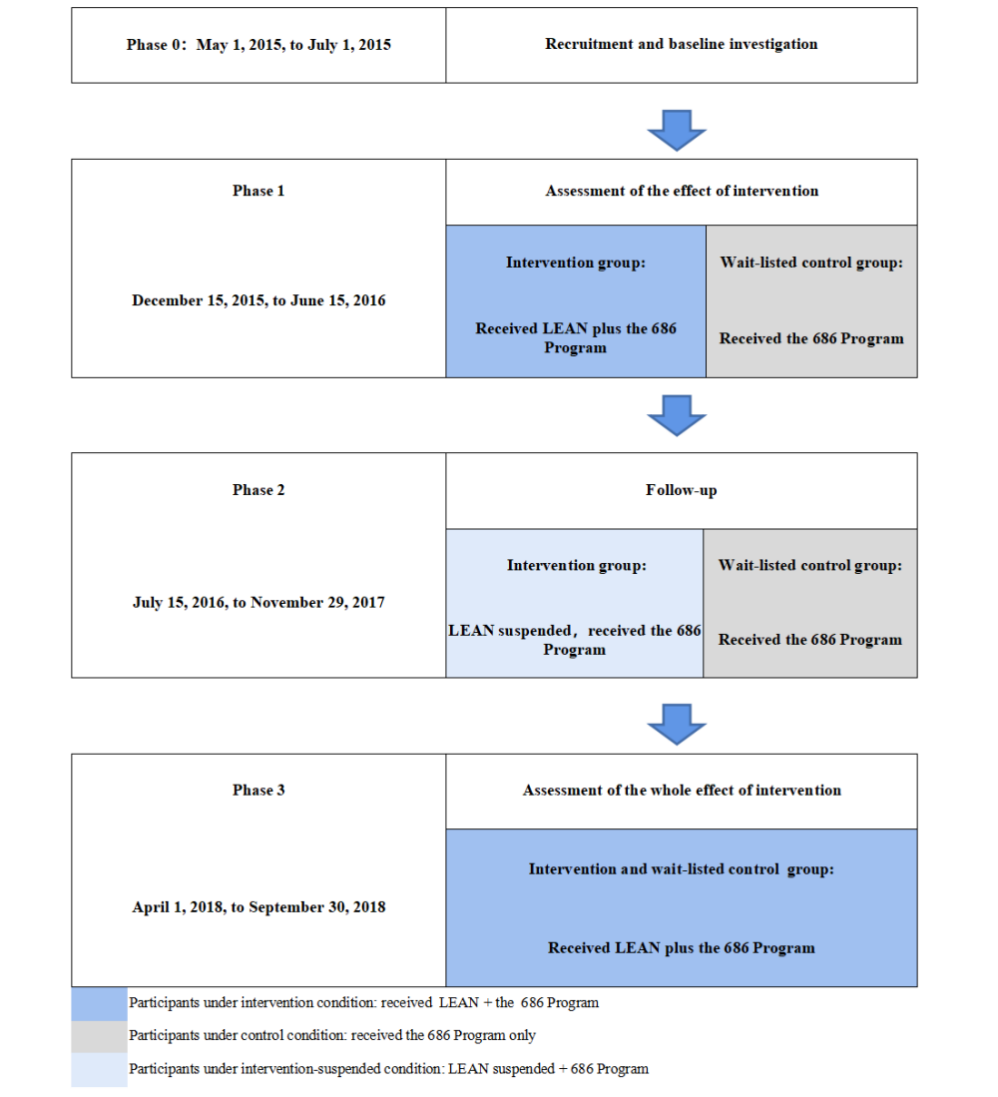
Study design [20]. LEAN: Lay health supporters, e-platform, award, and integration.

The whole program process was divided into 3 phases [20]. We conducted a 2-arm randomized controlled trial in phase 1. In the 6-month phase 1 (December 15, 2015, to June 15, 2016), the intervention group received LEAN plus the aforementioned 686 Program for 6 months and the control group received the 686 Program only (trial registration: Chinese Clinical Trial Registry ChiCTR-ICR-15006053) [16]. After that, the LEAN intervention was suspended, and both groups received the 686 Program only (phase 2: from July 15, 2016, to November 29, 2017). Then, we restarted our intervention and extended it to both the original intervention group and the control group, and all participants received the LEAN intervention in addition to the 686 Program from April 1, 2018, to September 30, 2018 (Figure 1). The results of phases 1 and 3 have been published elsewhere [6,20]. This study aims to examine the results of phase 2 (ie, the results at the 18th month after the suspension of LEAN).

### Procedures and Interventions

The development of our intervention *LEAN* was guided by the Health Belief Model [21-23], existing empirical evidence, and preliminary data from our pilot research [16]. On the basis of the Health Belief Model, patients’ uptake of a health service depends on their evaluation of the risks of the health problem, the perceived barriers to and benefits of the action, and a *cue* or stimulus for action. Accordingly, the acronym of our intervention LEAN included four elements [6]: (1) lay health supporters (often a designated family member to watch patient medication, side effects, relapses, and urgent care), (2) e-platform (texting system for medication reminders, health education, and relapse monitoring), (3) award (token gifts to encourage behavioral improvement), and (4) integration of the texting with the existing health system to enable collaborative care. The e-platform was the core of the LEAN intervention. The educational and monitoring messages were intended to help the patients and families recognize the harm of schizophrenia and the benefits of taking medications. The SMS text messaging reminders serve as a *cue* to urge the patients to take action—essentially taking the medication.

### Setting and Participants

The trial was conducted in a resource-poor setting: 9 rural townships of Liuyang municipality (population 356,900), Hunan province, China. We included the patient participants if they (1) were living in the community rather than being institutionalized at the time of our recruitment, (2) were enrolled in the 686 Program, (3) had a confirmed diagnosis of schizophrenia according to International Classification of Diseases 10th Revision [24], (4) took oral psychotropic medications, and (5) resided in one of the nine rural townships of Liuyang municipality. People were excluded if they (1) were hospitalized for schizophrenia at the time of recruitment (our approach was community-based), (2) had missed 3 consecutive refills (in this case, they had de facto dropped out of the 686 Program), or (3) were physically incapable of using voice or SMS text messaging (hearing or vision impairment prevented the use of our intervention). Using simple random sampling, we drew a representative sample from the registry of the 686 Program, which included almost all known people with schizophrenia in Liuyang.

### Outcomes

The details of the procedures to our outcome measurement and data collection were described in our published protocol and the earlier papers [6,16,20,25]. We provide a brief description in this section. We tracked all prespecified outcomes except the adherence measured by the Brief Adherence Rating Scale and the Drug Attitude Inventory-10 [16], which were not conducted as planned because of our errors in program implementation. The primary outcome was antipsychotic medication adherence as assessed by unannounced home-based pill counts (ie, the proportion of dosages taken in the past month). A total of 2 counts at a 30-day interval were performed to obtain the adherence using the following formula: (number of the first count − number of the second count + number of additional pills obtained − number of pills discarded) ÷ (number of pills prescribed). The count was considered *unannounced* as although the participants consented to the count, they were unaware of the specific timing of the count, which means that they were unaware of the first and second times of home visiting. The assessors (public health or medical students) were blinded to the group assignments and were rigorously trained to follow a pill count protocol that required inquiry on the number of purchased and discarded pills. The number of prescribed pills was abstracted from the 686 Program system. The pill count adherence was supplemented with medication refill adherence captured from the 686 Program system (number of refills required ÷ number of refills conducted over the past 6 months). We also measured two secondary outcomes: the patient’s functioning assessed with the World Health Organization Disability Assessment Schedule 2.0 (WHODAS) [26,27] and symptoms assessed using the Clinical Global Impression–Schizophrenia (CGI-Sch) measure [28]. The student assessors administered the WHODAS in an interview with the patients. The 686 Program psychiatrists performed symptom evaluations using CGI-Sch. The 686 Program system routinely tracked a host of other outcomes, including rehospitalization due to schizophrenia, death for any reason, suicide, and wandering. We also collected data on violence in the past 6 months. In addition, we assessed at baseline a few other empirically suggested strong predictors of medication adherence, including medication side effects (assessed using the self-administered Glasgow Antipsychotic Side-Effect Scale [29]), smoking and alcohol abuse (ie, substance abuse), and family supervision on medicine [30].

### Sample Size

The details of the sample size calculation were described by Xu et al [6,16]. Briefly, we calculated the sample size of 258 patient participants (129 per group) to ensure 85% power to detect the program effect of increasing the medication adherence from 0.72 to 0.85 (SD 0.33), which was considered minimally clinically acceptable difference after consultation with the 686 Program policy makers. The calculation assumed a 5% type I error and 10% attrition of participants.

### Allocation and Blinding

A statistician who was not otherwise associated with the project assigned the participants equally to the intervention or control group using simple randomization. It was impossible to blind the program participants regarding their group assignment, but the outcome assessors and psychiatrists were blinded to the allocation status. The assessors were physically separated from the LEAN implementation team as well. Unmasking was reported immediately, and a makeup assessment was scheduled with separate assessors [16].

### Statistic Methods

In this analysis, our primary objective was to analyze the sustained effect of LEAN at 18 months after the suspension of the LEAN intervention. Following our protocol, we used a generalized estimating equation model through the *gee( ) package* (gee; version 4.8) in R (RStudio; version 3.5.3) for statistical analyses. The generalized estimating equation model can provide a robust analysis of the abnormally distributed data of our primary outcome (adherence), which is the same as our phase1 analysis mode [6], so we can compare the difference between the 2 phases on the same model. Our primary outcome (adherence) analysis was adjusted for 7 covariates that were empirically suggested and prespecified baseline predictors of adherence in our protocol [16]. Multimedia Appendix 1 presents the primary and secondary analysis models and their equations.

We performed several sensitivity analyses to compare the results of the program’s effects on adherence, functioning, and symptoms by fitting an unadjusted analysis without data imputation. We also fit a model with multiple imputation (MI) but without adjusted covariates and a model with both adjusted analysis with covariates and MI for the missing data. The details of MI are presented in Multimedia Appendix 2. We conducted the same analysis with covariate adjustment for the two prespecified subgroups: a baseline nonadherent group (missing any of the previous 6 refills) and the group with low baseline functioning (WHODAS: cutoff at ≤0.22). We performed an intention-to-treat analysis for all participants [31]. The fully conditional specification, an MI method, was used to impute missing data [32]. We calculated the program effect size as Cohen *d* [33] to enable a cross-study comparison.

### Ethics and Dissemination

The study has obtained the institutional review board approval from the University of Washington (49464 G) and Central South University (CTXY-150002-6). All patient participants and their lay health supporters provided written informed consent.

### Patient and Public Involvement

Our study fully considered stakeholders’ suggestions during the study design and implementation phases. The patients and family members directly informed our intervention. We piloted a system of asking the village doctors to observe the patients for medication taking directly. However, we later completely dropped this initial idea after consulting patients and doctors for their experiences in the pilot. They felt this was very intruding and added a reasonable burden to the village doctors. Taking the advice from the families, we revised our original plan and developed LEAN that relied on family members and mobile texting to help the patients. The stakeholders also helped us refine the frequency of texting, the role of lay health supporters, how the rewards would be delivered, and how the intervention would be integrated with the existing 686 Program. During the implementation phase, we also regularly captured the feedback from the stakeholders and used the information to further refine the program.

### Protocol

The study design, methods, and analysis plan have previously been published as a study protocol [16].

## Results

### Participants

**Figure 2:**
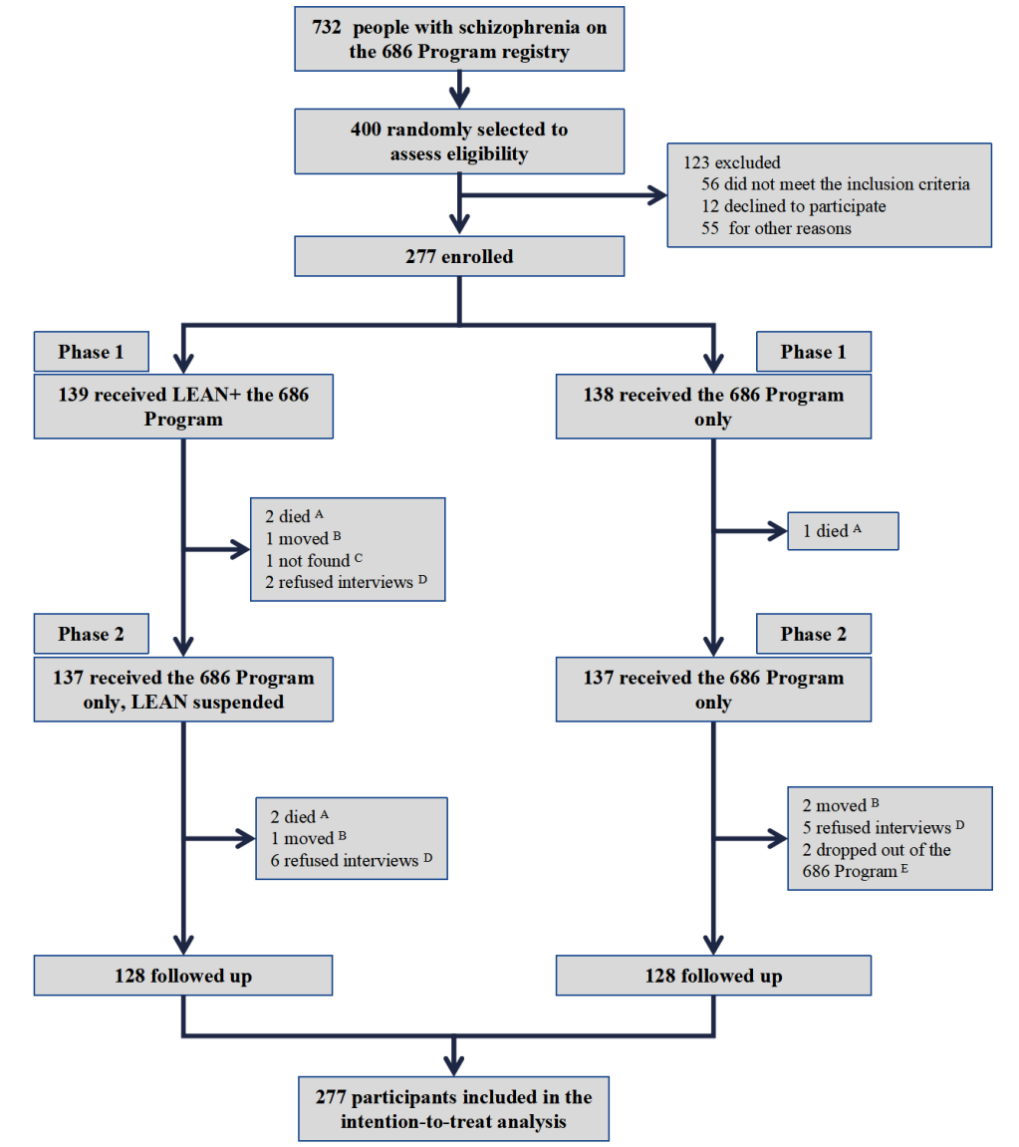
Participant flow [20]. (A) Died and thus no longer received the program; (B) moved but still received the program; (C) not found in the following interviews but still received the program; (D) refused interviews but still received the program; (E) dropped out of the program and thus no longer received the program. LEAN: Lay health supporters, e-platform, award, and integration.

We successfully recruited 277 patient participants (more than what we planned because of a higher level of interest): 139 randomized to the intervention group and 138 to the control group. Multimedia Appendix 3 summarizes the baseline characteristics between the intervention and control groups, suggesting no characteristic differences between the 2 groups. In phase 2, we collected the primary and secondary outcomes in 2 home visits conducted from November 24 to 29, 2017, and from December 24 to 29, 2017, respectively. The participants’ flow is presented in Figure 2.

### Retention

Both the intervention and control groups had 128 (256/277, 92.4%) participants who completed phase 2 follow-up for the full range of information through home-based visits. However, we captured the data of 98.2% (272/277) of the participants for medication refill adherence when they came to the township health center to refill medicine. Among the 256 participants followed up by the home visit or visit the township center, 166 (64.8%) were successfully interviewed for pill count adherence, 247 (95.5%) for functioning (WHODAS), and 256 (100%) for symptoms (CGI-Sch). The analyses of the missing data pattern and the results of MIs are presented in Multimedia Appendix 2. The major results of this study are summarized in Table 1.

**Table 1:** Outcomes of different groups and periods in each phase.^a^

Measures and phase					Intervention group^b^ (n=139)		Control group^b^ (n=138)		Mean difference or relative risk^c^ (95% CI)		*P* value
**Primary outcome, mean (SD)**											
	**Pill count adherence^d^**										
			1	0.61 (0.34)		0.48 (0.35)		0.11 (0.03 to 0.20)		.007	
			2^e^	0.62 (0.30)		0.58 (0.41)		0.05 (−0.06 to 0.16)		.41	
	**Other measurements**										
		**Refill record^f^**									
			1	0.83 (0.28)		0.76 (0.34)		0.08 (0.003 to 0.15)		.04	
			2	0.78 (0.34)		0.73 (0.37)		0.05 (−0.04 to 0.13)		.28	
		**DAI-10^g,h^**									
			1	0.68 (0.20)		0.67 (0.22)		0.01 (−0.05 to 0.07)		.75	
			2	N/A^i^		N/A		N/A		N/A	
		**BARS^j,k^**									
			1	0.71 (0.21)		0.68 (0.23)		0.03 (−0.03 to 0.08)		.38	
			2	N/A		N/A		N/A		N/A	
**Secondary outcome, mean (SD)**											
	**WHODAS^l,m^**										
			1	0.12 (0.15)		0.14 (0.19)		−0.02 (−0.07 to 0.01)		.22	
			2^e^	0.16 (0.17)		0.20 (0.22)		−0.03 (−0.08 to 0.02)		.19	
	**CGI-Sch^n^ severity of illness^o^**										
			1	2.84 (1.37)		2.76 (1.24)		0.12 (−0.19 to 0.44)		.44	
			2^e^	2.50 (1.08)		2.58 (1.17)		−0.02 (−0.29 to 0.24)		.86	
	**Negative**										
			1	2.61 (1.32)		2.85 (1.28)		−0.24 (−0.62 to 0.14)		.22	
			2	2.55 (1.04)		2.55 (1.13)		−0.01 (−0.27 to 0.26)		.97	
	**Positive**										
			1	2.62 (1.32)		2.85 (1.24)		−0.24 (−0.63 to 0.14)		.22	
			2	2.51 (1.08)		2.60 (1.18)		−0.08 (−0.36 to 0.19)		.55	
	**Depression**										
			1	2.25 (1.18)		2.06 (0.99)		0.19 (−0.13 to 0.51)		.25	
			2	2.60 (1.17)		2.65 (1.27)		−0.06 (−0.35 to 0.24)		.72	
	**Cognition**										
			1	2.66 (1.31)		2.90 (1.25)		0.19 (−0.13 to 0.51)		.25	
			2	1.99 (0.92)		2.02 (0.98)		−0.02 (−0.25 to 0.21)		.84	
	**CGI-Sch degree of change^p^**										
			1	3.09 (1.15)		3.02 (1.08)		0.04 (−0.23 to 0.32)		.76	
			2^e^	3.16 (1.13)		3.10 (1.20)		0.05 (−0.23 to 0.34)		.72	
**Other outcomes of the 686 Program^q^, n (%)**											
	**Rehospitalization due to schizophrenia**										
			1	9 (7.3)		25 (19.5)		0.37 (0.18 to 0.76)		.007	
			2	11 (8.4)		17 (12.9)		0.65 (0.32 to 1.33)		.24	
	**Dead for any reason**										
			1	2 (1.4)		1 (0.8)		1.99 (0.18 to 21.65)		.57	
			2	2 (1.6)		0 (0)		N/A		N/A	
	**Suicide**										
			1	0 (0)		0 (0)		N/A		N/A	
			2	1 (0.7)		0 (0)		N/A		N/A	
	**Wander**										
			1	2 (1.4)		2 (1.5)		1.00 (0.96 to 1.06)		.98	
			2	1 (0.7)		1 (0.7)		1.01 (0.98 to 1.02)		.99	
	**Hurting people or smashing objects**										
			1	2 (1.5)		6 (4.5)		0.37 (0.18 to 0.76)		.007	
			2	1 (0.7)		0 (0)		N/A		N/A	
	**Making trouble**										
			1	0 (0)		0 (0)		N/A		N/A	
			2	1 (0.7)		0 (0)		N/A		N/A	
	**Self-harm**										
			1	0 (0)		0 (0)		N/A		N/A	
			2	0 (0)		0 (0)		N/A		N/A	

^a^Phase 1 results are cited from a published paper [6].

^b^The numbers in phases 1 and 2 represent the number of participants with complete information in the intervention and control groups, respectively, at the end of that phase.

^c^Other than other outcomes of the 686 Program, all outcomes are presented as mean differences. Meanwhile, pill count adherence, WHODAS, CGI-Sch severity of illness, and CGI-Sch degree of change are from the adjusted analysis that adjusted for baseline covariates and used data imputation for the missing data; all other outcomes were raw analysis results.

^d^The proportion of antipsychotic dosage taken over the past month assessed by unannounced home-based pillar counts (possible range 0-1).

^e^The results were from adjusted analysis with covariates and data imputation.

^f^Refill record adherence is (number of days medication obtained over the past 182 days) ÷ (182 days).

^g^DAI-10: Drug Attitude Inventory-10.

^h^Drug Attitude Inventory adherence was originally from −10 to +10 (higher score=more positive attitude toward medication), which was rescaled to 0 to 1.

^i^N/A: not applicable.

^j^BARS: Brief Adherence Rating Scale.

^k^BARS is the self-reported percentage of dosages administered over the past month.

^l^WHODAS: World Health Organization Disability Assessment Schedule 2.0.

^m^WHODAS: percentage of functioning loss (possible range 0-1).

^n^CGI-Sch: Clinical Global Impression–Schizophrenia.

^o^CGI-Sch severity of illness: higher scores indicate worse symptoms (possible range 1-7).

^p^CGI-Sch degree of change: higher scores indicate less change (possible range 1-7).

^q^These outcomes were tracked by the 686 Program administrative system on a routine basis. A small number of data points were missing.

### Adherence Outcomes

**Figure 3:**
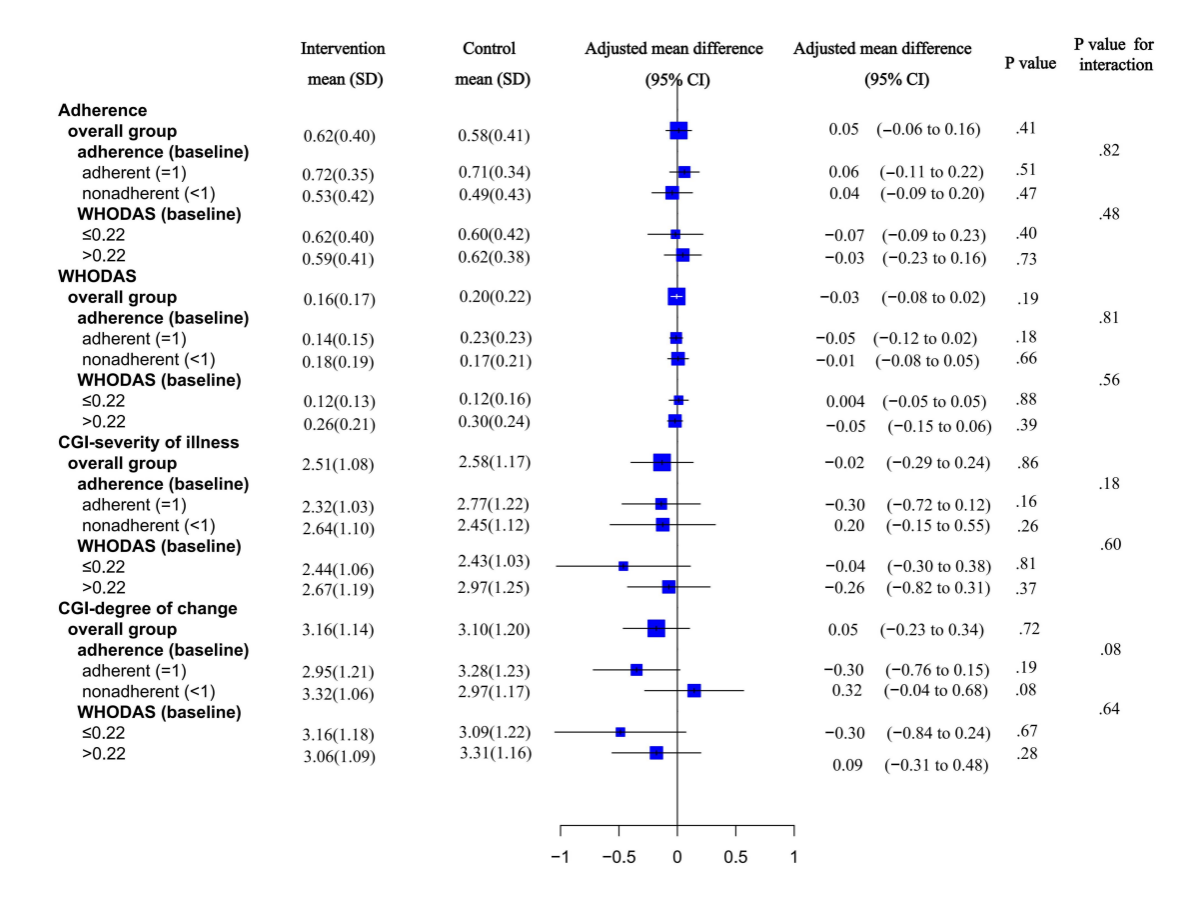
Subgroup analyses. CGI: Clinical Global Impression; WHODAS: World Health Organization Disability Assessment Schedule 2.0.

In our study, the top 3 prescriptions of antipsychotic medications in phases 1 and 2 remained the same (Multimedia Appendix 4). In phase 1, the antipsychotic medication adherence improved after the intervention. However, in phase 2, there was no statistical significance of the difference of the antipsychotic medication adherence on pill counts between the intervention and control groups. In phase 1, the antipsychotic medication adherence based on pill counts increased from 0.48 (SD 0.35) in the control group to 0.61 (SD 0.34) in the intervention group in the adjusted analysis (adjusted mean difference 0.11, 95% CI 0.03-0.20; *P*=.007; Cohen *d* effect size=0.38; Table 1) [6]. However, the phase 2 results of this study indicated that, after the intervention was suspended, the difference in adherence between the 2 groups narrowed and was no longer significant. However, this narrowing effect for the antipsychotic medication adherence was mainly due to an increase in adherence in the control group rather than a decrease in the intervention group (mean 0.62, SD 0.30 in the intervention group and mean 0.58, SD 0.45 in the control group; adjusted mean difference 0.05, 95% CI −0.06 to 0.16; *P*=.41; Cohen *d* effect size=0.11; Table 1). Subgroup analyses did not note significant improvement in phase 2 (Figure 3). We present the frequency of antipsychotic medication adherence and discontinuance reasons in phases 1 and 2. We also conducted a retrospective analysis of antipsychotic medication adherence in phase 1 for participants in phase 2. These results are presented in Multimedia Appendix 5, which shows that the antipsychotic medication adherence was statistically significantly different in the LEAN intervention group and control group in phase 1 (adjusted mean difference 0.12, 95% CI 0.01-0.21; *P*=.003).

### Functioning

In phase 1, the mean scores of functioning were 0.12 (SD 0.15) and 0.14 (SD 0.19). There was no improvement in functioning. We still observed no improvement in functioning in phase 2, which has mean scores of 0.16 (SD 0.17) and 0.20 (SD 0.22; adjusted mean difference −0.03, 95% CI −0.08 to 0.02; *P*=.19; Cohen *d* effect size=0.20; Table 1). There was no significant improvement in functioning severity for the prespecified subgroups in phase 2 either (Figure 3).

### Symptoms

In phase 1, there was no improvement in symptoms, with scores of CGI-Sch severity of illness of the 2 groups being 2.84 (SD 1.37) and 2.76 (SD 1.24). We did not note any improvement in CGI-Sch severity of illness, with scores between groups being 2.50 (SD 1.08) and 2.58 (SD 1.17; adjusted mean difference −0.02, 95% CI −0.29 to 0.24; *P*=.86; Cohen *d* effect size=0.07; Table 1), and CGI-Sch degree of change, with scores between groups being 3.16 (SD 1.13) and 3.10 (SD 1.20; adjusted mean difference 0.05, 95% CI −0.23 to 0.34; *P*=.72; Cohen *d* effect size=0.05; Table 1) in phase 2 either. There was no significant improvement in the symptoms for the prespecified subgroups in phase 2 either (Figure 3).

### Other Outcomes

In phase 1, the incidence of rehospitalization due to schizophrenia decreased from 19.5% (25/128) under the control arm to 7.3% (9/124) under the intervention arm (relative risk 0.32, 95% CI 0.18-0.76; *P*=.007, number needed to treat 8.15, 95% CI 4.86-25.19; Table 1). However, the relative risk in rehospitalization in the original intervention group increased and was no longer significant, which was, however, mainly due to the reduced incidence of rehospitalization in the control arm. The incidence in the intervention group was 8.4% (11/131) and 12.9% (17/131) in the control group (relative risk 0.65, 95% CI 0.32-1.33; *P*=.24; number needed to treat 21.83, 95% CI 8.30-34.69; Table 1).

There was 1 patient reported for wandering, 1 for hurting people or smashing objects, 1 for making troubles, and 2 for death during the whole follow-up period, including 1 who committed suicide.

### Sensitivity Analysis

Sensitivity analyses (Multimedia Appendix 6) showed that the results were not sensitive to the different analytical methods. The analyses of pill count adherence at various cutoff points also showed the same results—other cutoff points not affecting the results of the evaluation of the intervention after it was withdrawn (*P*≥.05).

## Discussion

### Principal Findings

This study examined the sustaining effect of LEAN, a 2-arm randomized controlled trial of an SMS text messaging intervention, 18 months after its withdrawal. In phase 1 (the 6-month LEAN intervention), both the antipsychotic medication adherence based on pill counts and rehospitalization due to schizophrenia significantly improved (adherence from 0.48 to 0.61 and rehospitalization from 19.53% to 7.26%, control vs intervention) [6]. However, in phase 2 (18 months after the LEAN intervention was withdrawn), although adherence and rehospitalization in the intervention group almost remained at their phase 1 level, the control group participants’ adherence improved, leading to the disappearance of the program advantage of LEAN plus the 686 Program versus the 686 Program alone. We did not observe any improvement in other outcomes or subgroups either.

Some studies have found that after the discontinuation of the texting intervention, the effect can last for a short or long time [17-19]. However, we should note several differences between our study and the other studies. We focused on people with schizophrenia who often have other cognitive impairment, whereas other studies targeted groups with better cognition, such as smokers, people with HIV, and adults with diabetes [17,18]. Meanwhile, compared with our work, many studies had shorter follow-up times, ranging from 8 weeks to 15 months after the intervention discontinuation [17-19]. The residual effect of LEAN can only be established by a significant mean difference in adherence between the initially assigned intervention group and the control group at the end point of the 18 months. The disappeared program advantage in our study was mainly due to the improvement in the control group rather than a diminishing adherence on the intervention group. We offer several speculations on the explanation of this seemingly puzzling result. First, some external conditions that affect medication adherence may have deferentially developed between the 2 groups. For instance, the intensity or quality of the 686 Program may have been applied differently to the 2 groups. However, our interviews with the families and program administrators did not support this. However, we cannot exclude other unknown factors. Second, there might have been a tortoise and hare effect [34] in that the discontinuation of texting reduced participants’ motivations in the intervention group to keep improving, whereas the participants in the control group continued their gradual improvement at their original pace possible because of the management from the 686 Program. Third, there might be a ceiling effect that made it difficult for the adherence in the intervention group to continue improving. Fourth, the primary mechanism of LEAN may come from the texted *cues* to take medications rather than a changed belief among the participants on the benefits of taking medicines versus cost. Once this reminder is stopped, the program effect may disappear, partly because of the potential cognitive impairment in our participants. Finally, we cannot exclude an alternative explanation that the initial program advantage in phase 1 results from chance. Thus, the earlier texting did not help the patients and families establish the new behavior of taking medicine. At the same time, the visit of the data collection team for pill count might serve as a reminder for the families and patients to adhere to medications to improve the adherence in both groups. However, the effect might be more robust in the control group when the families found the adherence was rather poor.

After phase 2, we resumed the LEAN intervention for both the original intervention and control groups (phase 3). The published results of phase 3 suggest LEAN’s effect on improving medication adherence over an extended implementation of LEAN (phase 3) [20]. No improvement in functioning has been observed throughout phases 1 [6], 2, and 3 [20]. In subgroup analyses, we did not find any functioning improvements in patients with different baseline adherence and functioning. We suspect the lack of effect was due to the ceiling effect. Because of the 686 Program that applied to both the intervention and control groups, people with schizophrenia in our study had much better functioning (WHODAS mean score 0.19) at baseline than other groups of people with schizophrenia of similar ethnicity and culture (WHODAS mean score ranging from 0.29 [35] to 0.64 [36]). People with schizophrenia in our study did not present a statistically significant improvement in symptoms between the intervention and control groups and in subgroups. However, we found a decreasing trend in CGI-Sch severity of illness scores and an increasing trend in CGI-Sch degree of change scores compared with themselves in phase 1. In phase 3, after an extended intervention, we found a statistically significant improvement in symptoms [20]. This indicates that sustained medication adherence may lead to improvement in symptoms.

Our study has several limitations, which may also point to future research directions. First, we should have conducted more systematic surveys and qualitative studies such as focus groups and interviews to understand participants’ experiences in both the 686 Program and LEAN to better analyze the improvement in adherence in the control group 18 months after the LEAN suspension. Second, our failure in conducting the planned Drug Attitude Inventory (due to a scheduling error) at the end point of the 18-month period further complicated our efforts to understand the evolution of the participants’ attitude toward the medications. Third, we did not systematically investigate the reasons for patients’ adherence and nonadherence. Some validated scales such as the Antipsychotic Discontinuation Questionnaire may be considered [37-39]. Fourth, although our rigor validated pill count methods [25], measurement errors were possible (eg, patients intentionally discarding pills may lead to inflated adherence measurement). However, this should not affect our program impact evaluation, as the behavior may occur for both groups and be balanced out because of our group randomization. Fifth, the low follow-up rate (166/277, 59.9%) for the primary adherence outcome was another limitation for our study. It resulted from the outcome-capturing method. We needed to interview the participants twice in 2 sequential months, and if there was 1 interview that failed, we could not count the difference of pills. If we informed the participants to wait for us at home, it would remind them to prepare for the pill counting, resulting in a false improvement in adherence. Finally, LEAN is a complex intervention because there were several program elements (ie, use of lay health supporters and program integration with the existing health system) besides the texted medication reminders. We cannot ascertain which component of the program delivered the largest effect. Neither can we test out the optimal dosage of texting in this trial. We strongly suggest future endeavors to consider trial designs proposed by implementation research, particularly those related to the Multiphase Optimization Strategy framework for the development of the complex intervention strategies and sequential multiple-assignment randomized trials [40] for the adaptive delivery of texting to people with schizophrenia [41]. Those new designs will refine the program elements of a complex intervention through systematically scheduled multiple phases and tailor the program intensity (dosage and contents of texting in this case) to different types of program participants. Quantitative comparative analysis may also be a valuable tool to understand the mechanism of the program [42]. In addition to these implementation research frameworks and methods, future studies may also use assessment frameworks for implementation outcomes such as Reach, Effectiveness, Adoption, Implementation and Maintenance to guide the implementation evaluation [43].

### Conclusions

Our results provide new evidence and research directions related to maintaining adherence to medication after the intervention was terminated. Our study is inclined to suggest that continuous prompts for medicine may be necessary to support medication adherence for people with schizophrenia. However, we cannot definitively exclude other alternative interpretations.

## Appendix

Multimedia Appendix 1 The analyses model and their equations in our study.

Multimedia Appendix 2 Multiple imputation used in our statistical analysis.

Multimedia Appendix 3 Baseline characteristics of participants.

Multimedia Appendix 4 Prescribed antipsychotic medications in phases 1 and 2.

Multimedia Appendix 5 Antipsychotic medication adherence and discontinuance reasons in phases 1 and 2.

Multimedia Appendix 6 Sensitivity analysis for phase 2 outcomes.

## Abbreviations

LEAN: Lay health supporters, e-platform, award, and integration
CGI-Sch: Clinical Global Impression–Schizophrenia
MI: multiple imputation
WHODAS: World Health Organization Disability Assessment Schedule 2.0
