# Supplementary material for: Residual Effect of Texting to Promote Medication Adherence for Villagers with Schizophrenia in China: 18-Month Follow-up Survey After the Randomized Controlled Trial Discontinuation
Source: JMIR Mhealth Uhealth. 2022 Apr 19;10(4):e33628. doi: 10.2196/33628 (PMC9066323; doi:10.2196/33628)
Supplement: Multimedia Appendix 1 [file mhealth_v10i4e33628_app1.docx]

### Appendix 1 The analyses model and their equations in our study

The analysis of our primary outcome (adherence) was adjusted for seven covariates that were empirically suggested and pre-specified baseline predictors of adherence in our protocol. In this model [equation (1)], we can succinctly write the model as:

$Y_{i}=\beta_{0}+\beta_{t}x_{t}+\beta_{1}x_{1}+{\beta_{2}x}_{2}+\beta_{3}x_{3}+\beta_{4}x_{4}+\beta_{5}x_{5}+\beta_{6}x_{6}+\beta_{7}x_{7}+\epsilon_{i}$ (1)

where $\epsilon_{i}～i.i.d.(0,\sigma^{2})$ and assumes a normal parametric model but is robust to misspecification. $\beta_{0}$ was the model intercept and $\beta_{t,1～7}$ were the regression coefficients of each independent variable, each $x$ was the independent variable. Where $x_{t}$ was the treatment. When $x_{t}=1$ means receive LEAN plus 686 Program, $x_{t}=$0 means receive 686 Program only. The $x_{1}$was baseline adherence, which was obtained from the refill records; the $x_{2}$ was the overall severity of illness and the $x_{3}$ was negative symptoms, which were obtained from the CGI-Sch scores; the $x_{4}$ was functioning, which was obtained from the WHODAS scores; and the$x_{5}$ was substance use, the $x_{6}$ was medication side effects, and the $x_{7}$ was family supervision which was obtained from the face-to-face interviewer when the patients and/or their caregivers came to refill medicine in the township health center. All covariates used for adjustment were measured at baseline, which were captured before the LEAN intervention was implemented.

The analyses of the secondary outcomes (WHODAS and CGI-Sch-severity of illness and CGI-Sch-degree of change) were adjusted by their baseline information [equation (2)], which was the same as the GEE model in phase 1. We can succinctly write the model as:

$Y_{i}=\beta_{0}+\beta_{t}x_{t}+\beta_{1}x_{1}+\epsilon_{i}$ (2)

where each *β* was the regression coefficient of each independent variable, and each $x$ was the independent variable. Where $x_{t}$ was the same as equation (1). The$x_{1}$was baseline scores of WHODAS or CGI-Sch-severity of illness or CGI-Sch-degree of change.
