# Supplementary material for: Residual Effect of Texting to Promote Medication Adherence for Villagers with Schizophrenia in China: 18-Month Follow-up Survey After the Randomized Controlled Trial Discontinuation
Source: JMIR Mhealth Uhealth. 2022 Apr 19;10(4):e33628. doi: 10.2196/33628 (PMC9066323; doi:10.2196/33628)
Supplement: Multimedia Appendix 2 [file mhealth_v10i4e33628_app2.docx]

### Appendix 2 Multiple imputation used in our statistical analysis

In the analysis process, we mainly use the R package mice, gee, norm. (MICE: Multivariate Imputation by Chained Equations). In the multiple imputations, we imputed 10 complete datasets. The GEE model is then applied for each dataset, and the systemization of 10 GEE model results is done with the norm R package. We describe the specific steps below, using the analysis of the effect of the program on medication adherence:

1. Assessing the missing data pattern: We visualized the pattern of missing data to discern any systematic pattern of missing (Fig 1). As we did not find any clear pattern, we assume that data are missing at random(MAR) in our analysis.

2. Selecting the data imputation model: If the variable with missing value is continuous, the predictive mean matching (PMM) method is used; if the variable with missing value is binary, Logistic regression is then used; while for a multi-level categorical missing variable, the polytomous regression is applied.

3. Determining the independent variables used in the imputation model: all other variables except for the dependent variable were used in the imputation model. To improve the accuracy of the imputation, in addition to the variables used for the primary GEE model including the variables of intervention assignment, adherence, functioning, substance use, drug side effects, family supervision, we also used other social-economic and demographic variables such as sex, age, education, income, living alone, duration of illness, symptoms severity, etc. in the imputation model.

4. Determining the order of imputation: From left to right: demographic/social-economic information and other variables, the independent variables used in the GEE model, dependent variables used in the GEE model.

5. Ten complete datasets generated were generated: Fig 3 shows the original observed data and the imputed data. The imputed data sets show a similar pattern of the data distribution to the original dataset.

6. The primary GEE model was performed for each of the 10 complete data sets.

7. Using the norm R package to synthesize the results from the 10 GEE models to obtain the overall estimate of the effect of the program.

References: MICE: Multivariate Imputation by Chained Equations in R

| 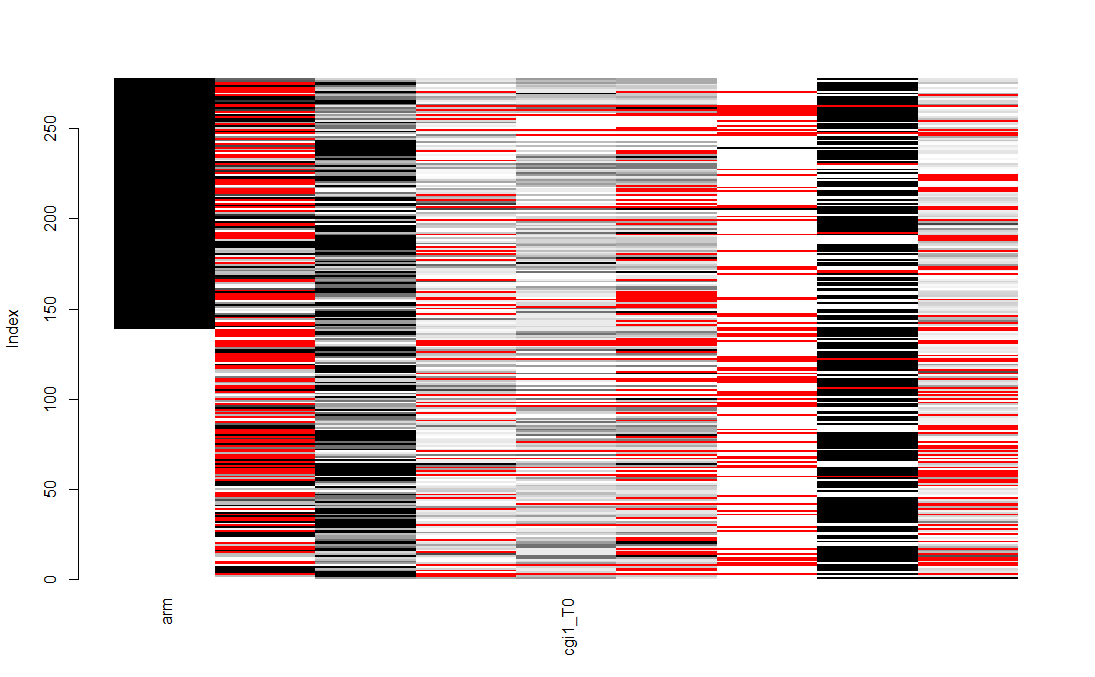  (a) | 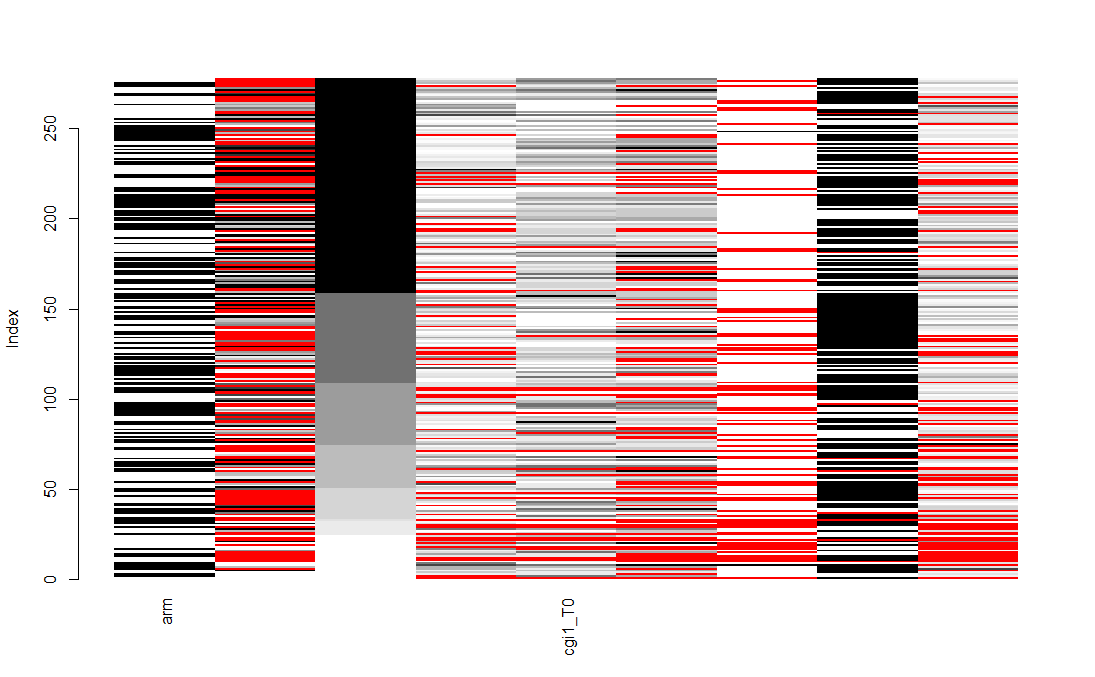  (b) |
| --- | --- |
| 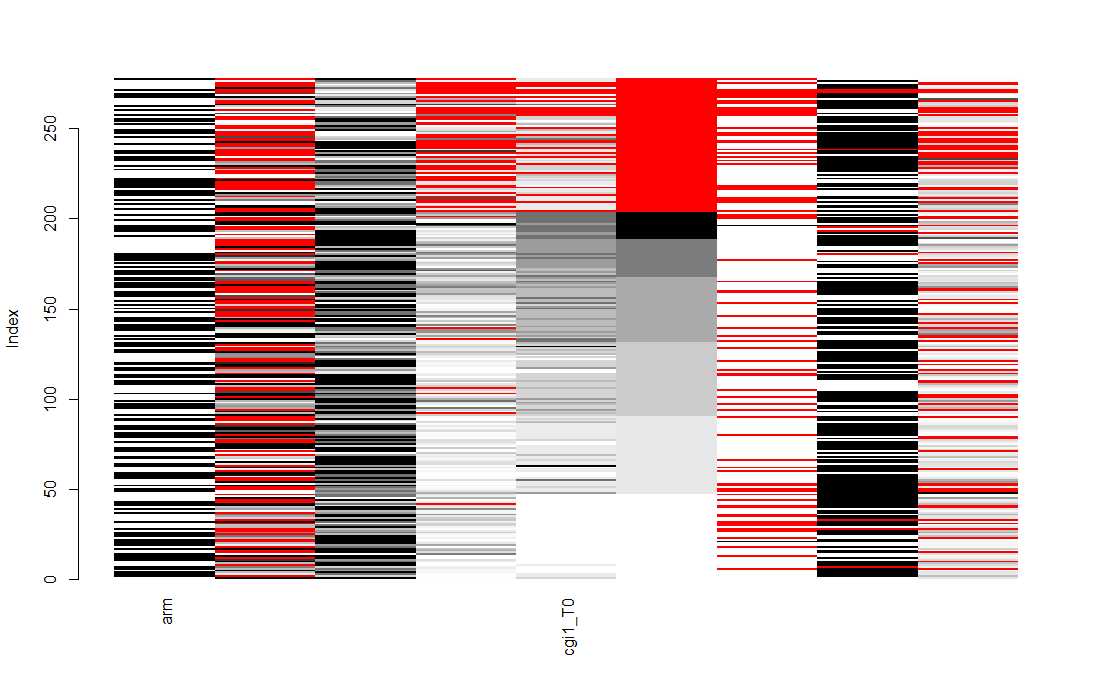  (c) | 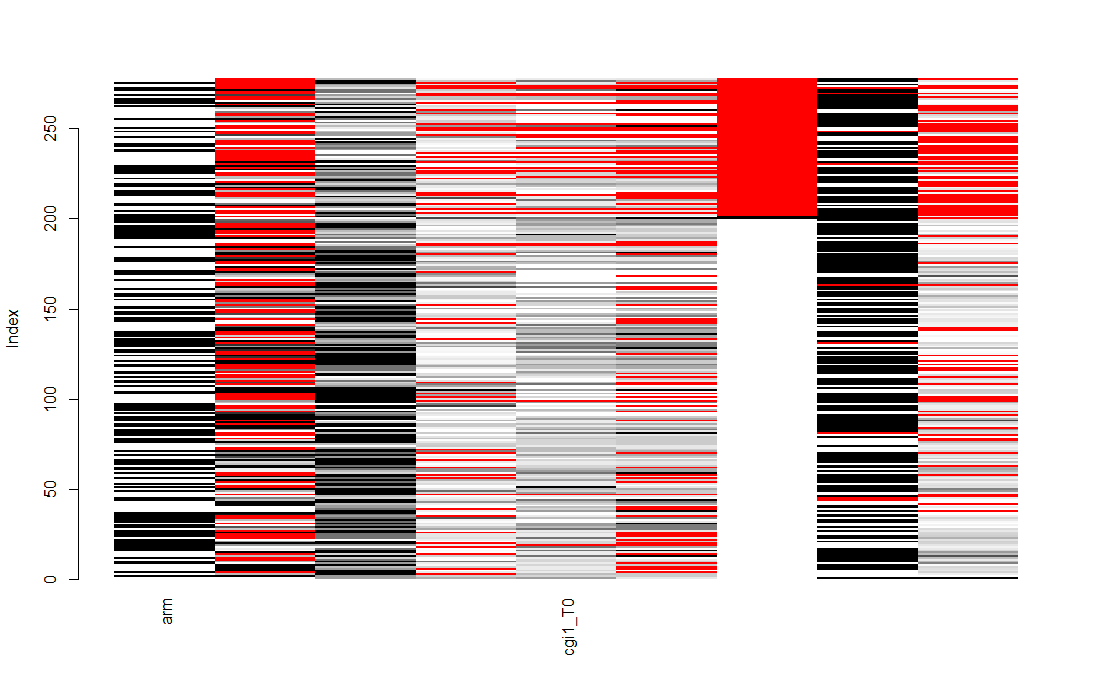  (d) |
| 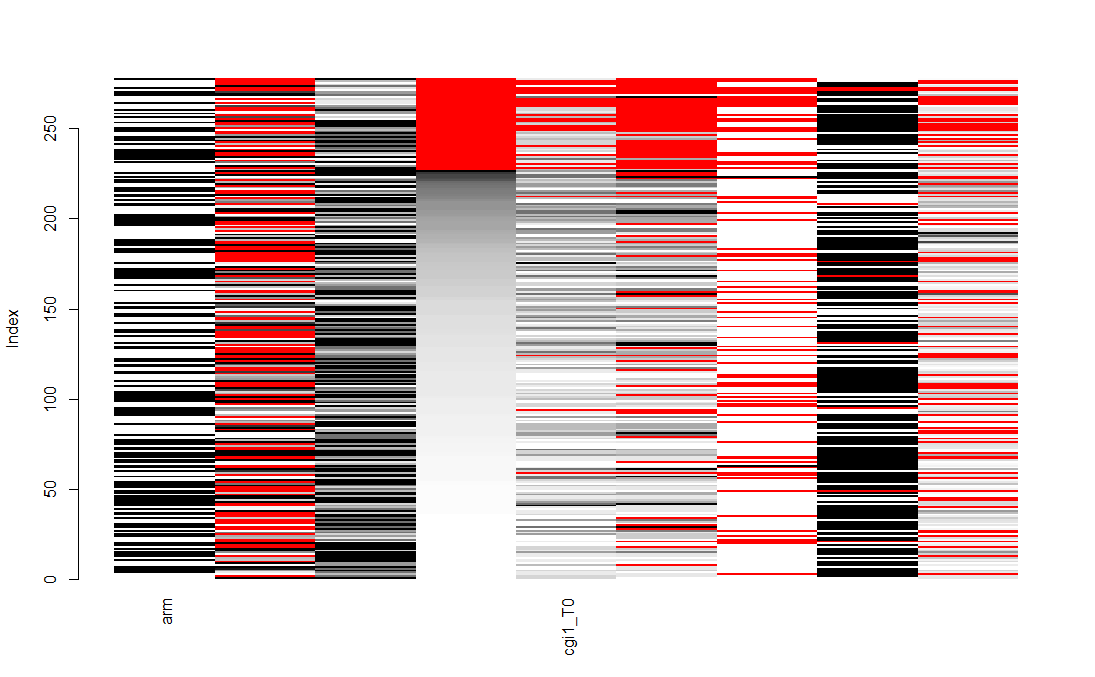  (e) | 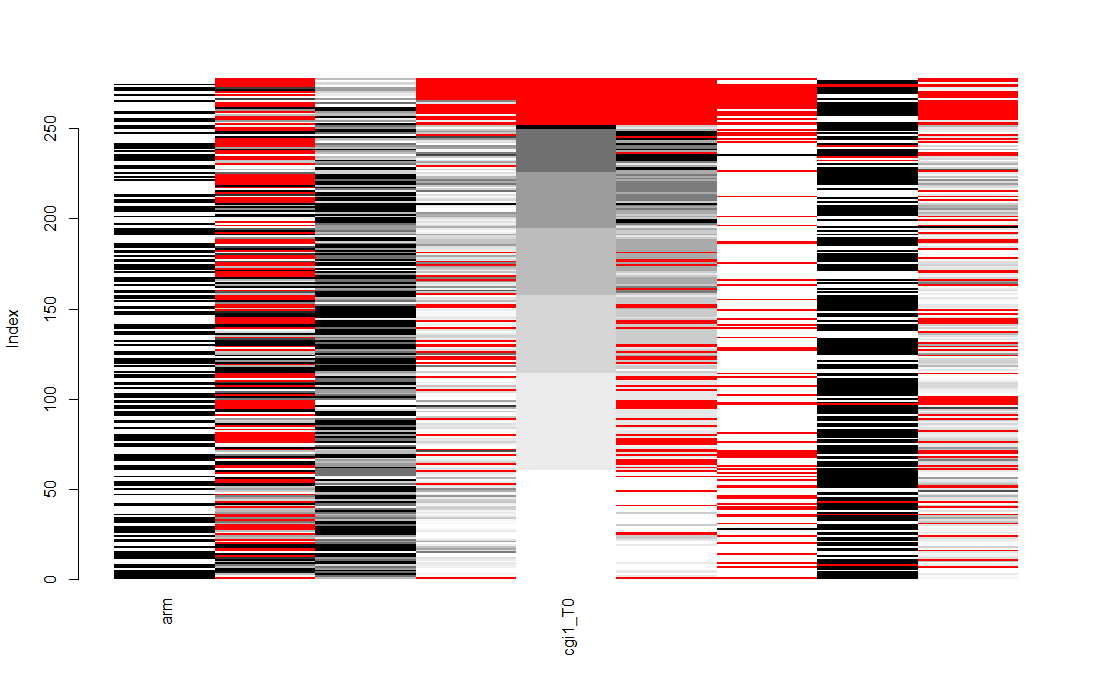  (f) |
| 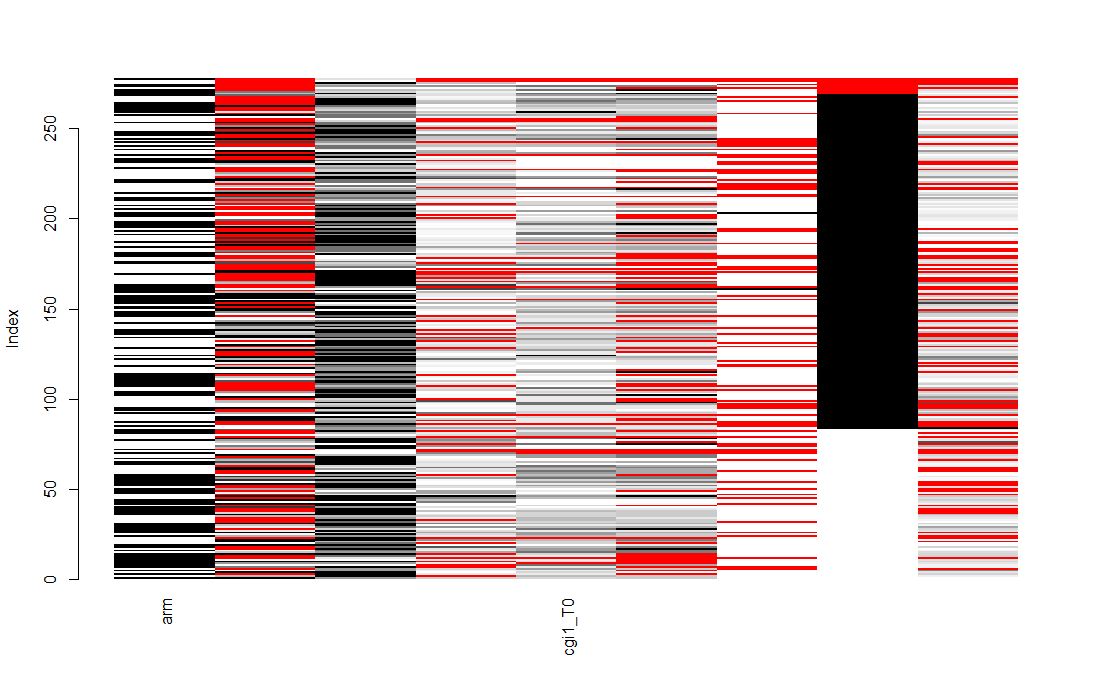  (g) | 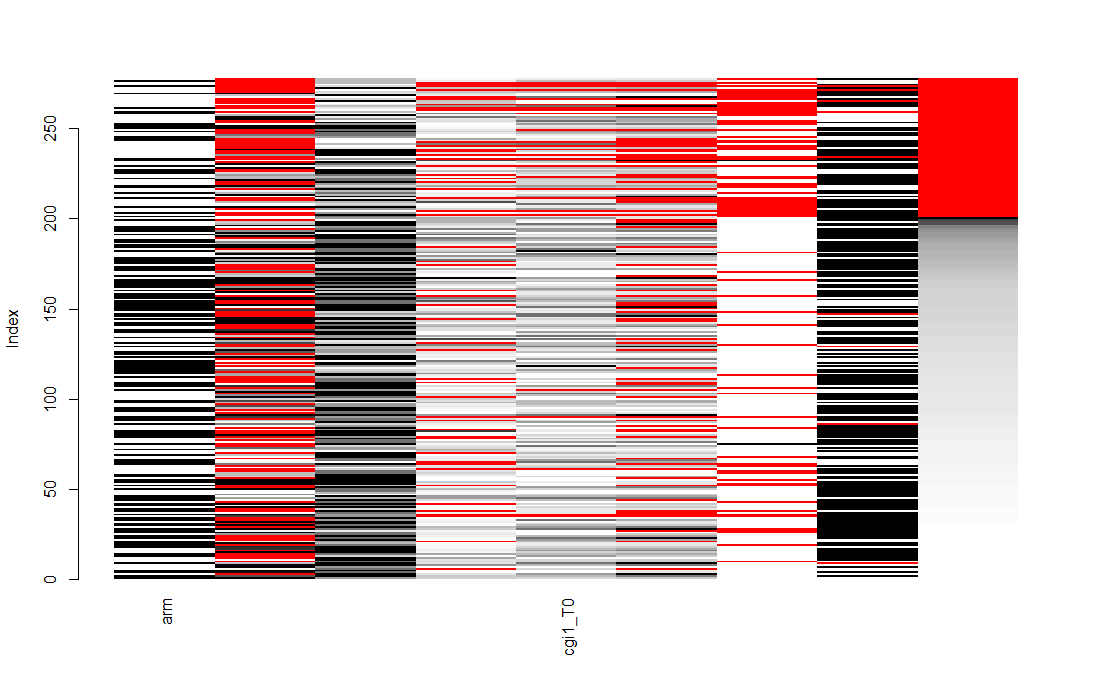  (h) |

Figure 1. Missing data description

Note : Sorted in order of (a)control vs. intervention condition; pre-specified baseline predictors: (b) pharmacy record; (c) functioning; (d)the overall severity of illness; (e)negative symptoms; (f)substance use; (g) medication side effects, and (h) family supervision. Red indicating missing; gray and black indicating available data; darker color indicating greater value.


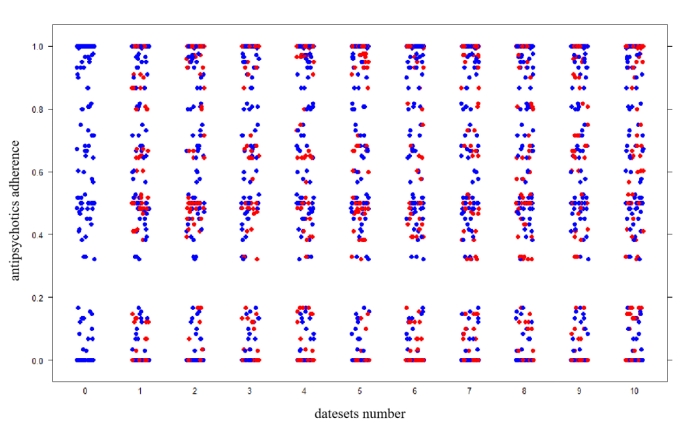


Figure 2. Imputed datasets

Note: Using antipsychotics adherence as an example; 0 represents the original observed data; 1-10 represent datasets after multiple imputations, with the blue dots indicating the original observed values, the red dots representing the imputed values
