# Supplementary material for: Residual Effect of Texting to Promote Medication Adherence for Villagers with Schizophrenia in China: 18-Month Follow-up Survey After the Randomized Controlled Trial Discontinuation
Source: JMIR Mhealth Uhealth. 2022 Apr 19;10(4):e33628. doi: 10.2196/33628 (PMC9066323; doi:10.2196/33628)
Supplement: Multimedia Appendix 3 [file mhealth_v10i4e33628_app3.docx]

### Appendix 3 Baseline characteristics of participants

Table 1. Baseline characteristics ^A^

| Baseline Characteristic | Intervention(n=139) | Control(n=138) |
| --- | --- | --- |
|  | Count(%) or Mean(SD) | Count(%) or Mean(SD) |
| **Patients** |  |  |
| Female | 77(55.4%) | 77(55.4%) |
| Married | 87(62.6%) | 90(64.8%) |
| Employed | 44(31.7%) | 48(34.5%) |
| Living alone | 7(5.0%) | 6(4.3%) |
| Age (years) | 46.5(12.7) | 45.5(12.7) |
| Education (years) | 7.4(3.3) | 7.1(3.2) |
| Literate ^B^ | 121(87.1%) | 126(90.6%) |
| Patient income last month (RMB) ^C^ | 66(0-500) | 95(0-800) |
| Family annual income (RMB ) ^C^ | 20,000(10,000-50,000) | 20,000(10,000-50,000) |
| Duration of Schizophrenia (years) | 17.5(10.4) | 18.37(10.8) |
| **Caregivers/Lay health supporters ^D^** |  |  |
| Female | 67(48.2%) | 67(48.2%) |
| Age (years) | 45.4(12.8) | 44.5(12.5) |
| Employed | 76(54.7%) | 62(44.6%) |
| Family members of the patient | 112(80.6%) | 109(78.4%) |
| Married | 77(55.4%) | 76(54.7%) |
| **Patients’ Health Profile** |  |  |
| Medication Adherence ^E^ |  |  |
| Refill record ^F^ | 0.8(0.3) | 0.7(0.4) |
| Drug Attitude Inventory (DAI) ^G^ | 0.7(0.2) | 0.7(0.2) |
| Brief Adherence Rating Scale (BARS) ^H^ | 0.7(0.2) | 0.7(0.2) |
| Clinical Global Impression-Severity (CGI) ^I^ | 2.9(1.7) | 3.1(1.7) |
| WHO Disability Assessment Schedule (WHODAS) ^J^ | 0.2(0.2) | 0.2(0.2) |
| Glasgow Antipsychotic Side-effect Scale (GASS) ^K^ | 9.57(6.7) | 8.6(8.0) |
| Top 5 Antipsychotics Prescribed |  |  |
| Clozapine | 48/136(35.3%) | 45/133(33.8%) |
| Risperidone | 46/136(33.8%) | 43/133(32.3%) |
| Quetiapine | 26/136(19.1%) | 25/133(18.8%) |
| Sulpiride | 21/136(15.4%) | 25/133(18.8%) |
| Perphenazine | 12/136(8.8%) | 15/133(11.3%) |

Note:

A: Cited from published paper[6]

B: Literate: Defined 3 or more years of primary school education.

C: Indicated as Median (IQR).

D: For the intervention group, those caregivers were recruited as “lay health supporters”.

E: Per our research protocol, medication adherence measured by unannounced home pill counts at endpoint was used for the analysis of program effect; however, pill counts were not performed at baseline. Instead, refill records and two rating scales were used at baseline.

F: Adherence by refill record was calculated as a cumulative medication possession ratio (0–100%) over a year, i.e., (# of days medication obtained over 365 days) ÷ (365 days).

G: DAI adherence was originally from −10 to +10 (higher score= more positive attitude toward medication), which was rescaled to be 0 to 1.

H: BARS adherence is the self-reported percentage of dosages taken over the past month.

I: Higher scores of CGI indicate worse symptoms (possible range 1–7).

J: WHODAS scores indicate the percentage of functioning lost.

K: GASS scores indicate patient-reported side effects of antipsychotics: scores 0-21 No/Mild side effects; 22-42 Medium side effects; 43 and above Serious side effects.
