# Supplementary material for: Residual Effect of Texting to Promote Medication Adherence for Villagers with Schizophrenia in China: 18-Month Follow-up Survey After the Randomized Controlled Trial Discontinuation
Source: JMIR Mhealth Uhealth. 2022 Apr 19;10(4):e33628. doi: 10.2196/33628 (PMC9066323; doi:10.2196/33628)
Supplement: Multimedia Appendix 4 [file mhealth_v10i4e33628_app4.docx]

**Appendix 4** **Prescribed antipsychotics in phase 1 and phase 2**

Table Prescribed antipsychotics in phase 1 and phase 2

| **ARM** | **Prescribe ^a^** | **Phase 1** | | | **Phase 2** | | |
| --- | --- | --- | --- | --- | --- | --- | --- |
|  |  | **n** | **N'** | **%** | **n** | **N'** | **%** |
| **Wait-list group** | Clozapine | 45 | 133 | 33.8 | 39 | 121 | 32.2 |
|  | Risperidone | 43 | 133 | 32.3 | 39 | 121 | 32.2 |
|  | Quetiapine | 25 | 133 | 18.8 | 16 | 121 | 13.2 |
|  | Sulpiride | 25 | 133 | 18.8 | 16 | 121 | 13.2 |
|  | Perphenazine | 15 | 133 | 11.2 | 7 | 121 | 5.8 |
|  | Ripiprazole | 12 | 133 | 9.0 | 9 | 121 | 7.4 |
|  | Olanzapine | 7 | 133 | 5.2 | 0 | 121 | 0.0 |
|  | Chlorpronmazine | 4 | 133 | 3.0 | 2 | 121 | 1.7 |
|  | Perphenazine [Injection] | 2 | 133 | 1.5 | 3 | 121 | 2.5 |
|  | Haloperidol | 1 | 133 | 0.8 | 1 | 121 | 0.8 |
|  | Penfluridol | 1 | 133 | 0.8 | 0 | 121 | 0.0 |
|  | Haloperidol Decanoate [Injection] | 0 | 133 | 0.0 | 0 | 121 | 0.0 |
|  |  |  |  |  |  |  |  |
| **Intervention group** | Clozapine | 48 | 136 | 35.3 | 41 | 125 | 32.8 |
|  | Risperidone | 46 | 136 | 33.8 | 41 | 125 | 32.8 |
|  | Quetiapine | 26 | 136 | 19.1 | 22 | 125 | 17.6 |
|  | Wulpiride | 21 | 136 | 15.4 | 14 | 125 | 11.2 |
|  | Perphenazine | 12 | 136 | 8.8 | 9 | 125 | 7.2 |
|  | Aripiprazole | 9 | 136 | 6.6 | 5 | 125 | 4.0 |
|  | Perphenazine [Injection] | 7 | 136 | 5.2 | 3 | 125 | 2.40 |
|  | Olanzapine | 5 | 136 | 3.7 | 0 | 125 | 0.0 |
|  | Ripiprazole | 5 | 136 | 3.7 | 4 | 125 | 3.2 |
|  | Penfluridol | 3 | 136 | 2.2 | 0 | 125 | 0.0 |
|  | Haloperidol | 2 | 136 | 1.5 | 0 | 125 | 0.0 |
|  | Trifluoperazine | 1 | 136 | 0.7 | 0 | 125 | 0.0 |
|  | Haloperidol Decanoate [Injection] | 0 | 136 | 0.0 | 0 | 125 | 0.0 |

^a.^ Those outcomes were tracked by the 686 Program administrative system on a routine basis.
