# Supplementary material for: Residual Effect of Texting to Promote Medication Adherence for Villagers with Schizophrenia in China: 18-Month Follow-up Survey After the Randomized Controlled Trial Discontinuation
Source: JMIR Mhealth Uhealth. 2022 Apr 19;10(4):e33628. doi: 10.2196/33628 (PMC9066323; doi:10.2196/33628)
Supplement: Multimedia Appendix 5 [file mhealth_v10i4e33628_app5.docx]

**Appendix 5 Antipsychotics adherence and discontinuance reasons in phase 1 and 2.**

Further analysis of adherence suggested that the number of the participants with perfect adherence (those never missing an antipsychotic dosage) increased from 40 [15.89% (17/107) in the control group, 20.72% (23/111) in the intervention group] at the end of phase 1 to 58 in phase 2 [36.36% (28/77)in the control group, 33.71% (30/89) in the intervention group]. The numbers of zero adherence (those never taking antipsychotics) increased from 26 [13.08% (14/107) in the control group, 10.81% (12/111) in the intervention group] in phase 1 to 33 [24.68% (19/77) in control and 15.73% (14/89) in intervention] in phase 2 (figure 1, a and b).

We conducted a retrospective analysis of antipsychotic adherence in phase 1 for participants in phase 2 (figure 1, c). Their antipsychotics adherence was statistical significantly different in the LEAN intervention group and control group in phase 1([AMD] 0.12 [95% CI: 0.01 to 0.21], *P=*0.0030) （Table 1）. This result indicated that the advantage of LEAN intervention would disappear when the intervention was suspended.

In phase 1, we did not record the discontinuance reasons. In phase 2, a few participants reported the discontinuance reasons (Table 2), however, we did not capture the reasons systematically.

| 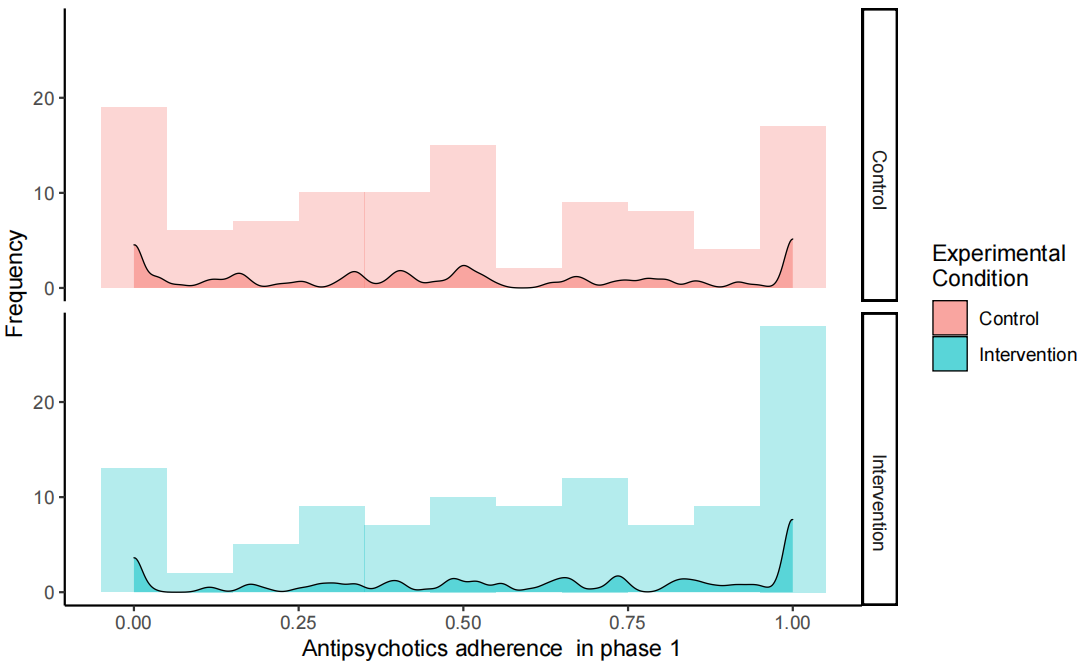  (a) |
| --- |
| 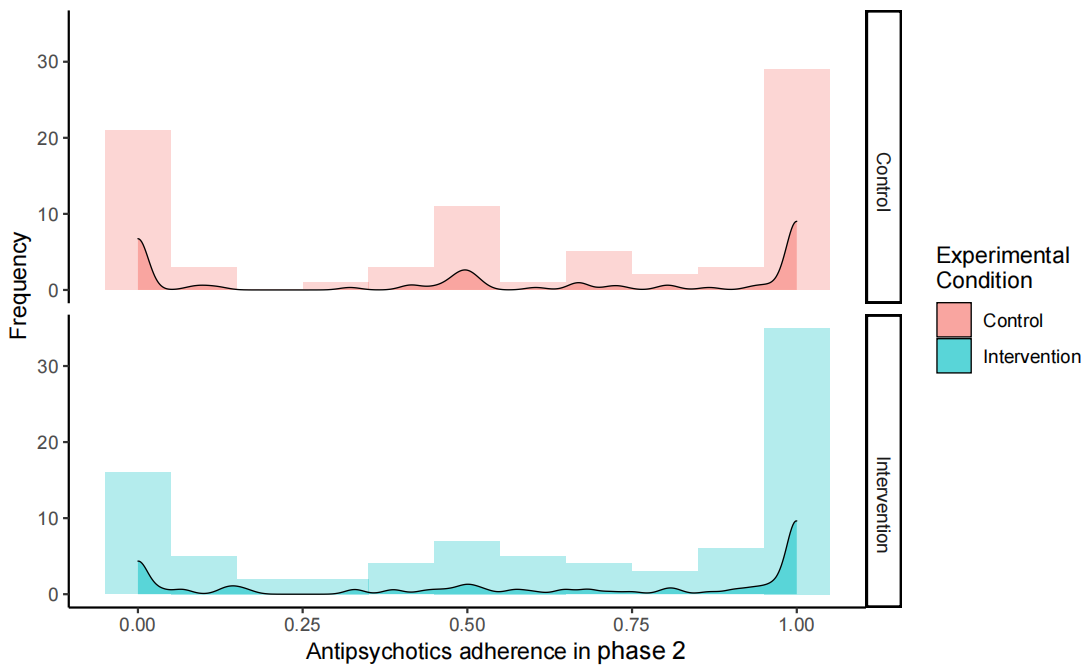  (b) |
| 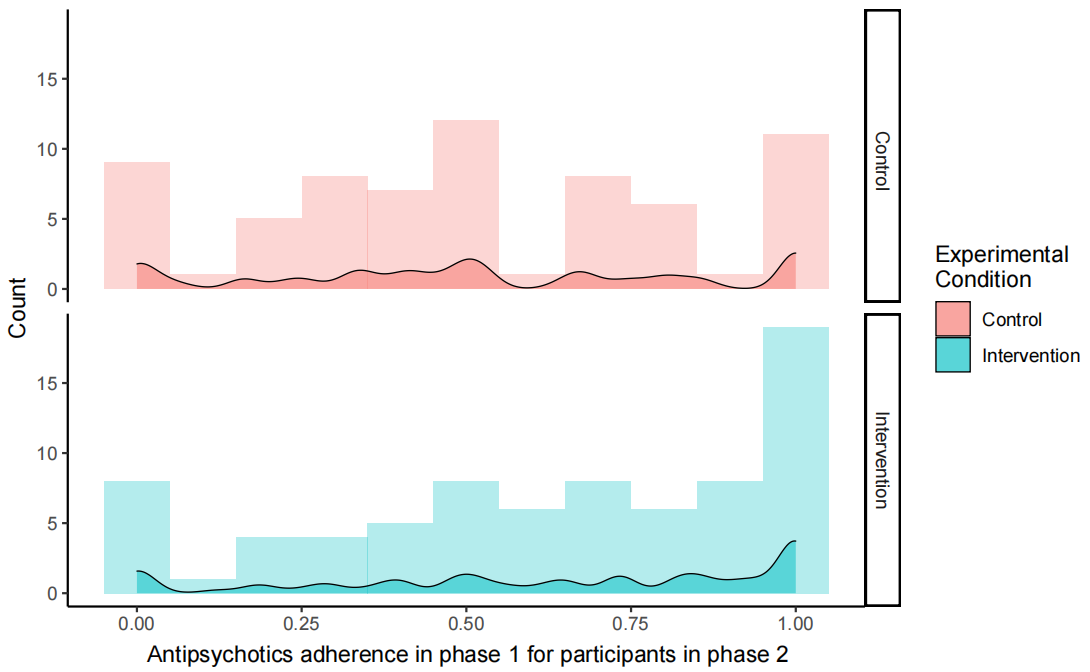  (c) |

Figure 1. Frequency of Antipsychotics Adherence in phase 1 and 2.

Table 1. The antipsychotics adherence in phase 1 for followed-up participants in phase 2

| **Measures** | **mean difference** | **95% CI** | | **P value** |
| --- | --- | --- | --- | --- |
| Treatment ^A^ | 0.119 | 0.011 | 0.227 | 0.030 |
| WHODAS | -0.260 | -0.603 | 0.083 | 0.140 |
| CGI | -0.048 | -0.120 | 0.023 | 0.185 |
| Negative-CGI | 0.05 | -0.028 | 0.128 | 0.205 |
| Substance-Abuse | -0.179 | -0.836 | 0.478 | 0.574 |
| GASS | -0.003 | -0.010 | 0.005 | 0.494 |
| Supervised | 0.076 | -0.040 | 0.193 | 0.201 |
| Pharmacy_record | 0.005 | -0.184 | 0.193 | 0.962 |

Note: Adjusted for baseline adherence (pharmacy record), the symptoms, as well as negative symptoms, functioning, substance use, medication side effects, and family supervision. The analysis was based on GEE model and multiple imputation.

Table 2. The Discontinuance Reasons for each group in phase 2

| **Discontinuance Reasons** | **intervention group** | | **control group** | |
| --- | --- | --- | --- | --- |
|  | **N'=14** | | **N'=19** | |
|  | **n** | **%** | **n** | **%** |
| Discontinuance without capture reasons | 13 | 92.86 | 14 | 73.68 |
| Feel alleviated | 0 | 0.00 | 3 | 15.79 |
| Refuse to take antipsychotic | 1 | 7.14 | 1 | 5.26 |
| Refuse to take antipsychotic due to side-effects | 0 | 0.00 | 1 | 5.26 |
| Discontinuance due to pregnancy | 0 | 0.00 | 1 | 5.26 |
