# Supplementary material for: Residual Effect of Texting to Promote Medication Adherence for Villagers with Schizophrenia in China: 18-Month Follow-up Survey After the Randomized Controlled Trial Discontinuation
Source: JMIR Mhealth Uhealth. 2022 Apr 19;10(4):e33628. doi: 10.2196/33628 (PMC9066323; doi:10.2196/33628)
Supplement: Multimedia Appendix 6 [file mhealth_v10i4e33628_app6.docx]

**Appendix 6 Sensitivity analysis for phase 2 outcomes**

Table 1. Raw versus Adjusted Analysis with covariates and Data imputation for phase 2 outcomes

| **Measures** | **Raw analysis** | | | |  | **Unadjusted Analysis based on imputation data** | | | | |  | **Adjusted Analysis based on imputation data** | | | |
| --- | --- | --- | --- | --- | --- | --- | --- | --- | --- | --- | --- | --- | --- | --- | --- |
|  | **mean difference** | **95%CI** | | **P value** |  | **mean difference** | **95%CI** | | **P value** | |  | **mean difference** | **95%CI** | | **P value** |
| Pill-count adherence | 0.04 | -0.08 | 0.16 | 0.510 |  | 0.06 | -0.06 | 0.17 | | 0.338 |  | 0.05^A^ | -0.06 | 0.16 | 0.410 |
| WHODAS | -0.04 | -0.09 | 0.01 | 0.145 |  | -0.04 | -0.09 | 0.01 | | 0.123 |  | -0.03^B^ | -0.08 | 0.02 | 0.191 |
| CGI-severity of illness | -0.08 | -0.35 | 0.20 | 0.591 |  | -0.04 | -0.31 | 0.23 | | 0.765 |  | -0.02^C^ | -0.29 | 0.24 | 0.863 |
| CGI-degree of change | 0.06 | -0.23 | 0.34 | 0.696 |  | 0.07 | -0.22 | 0.35 | | 0.649 |  | 0.05^D^ | -0.23 | 0.34 | 0.721 |

Note:

A: Adjusted for baseline adherence (pharmacy record), the symptoms, as well as negative symptoms, functioning, substance use, medication side effects, and family supervision.

B: Adjusted for baseline functioning.

C: Adjusted for baseline severity of illness.

D: Adjusted for baseline degree of change.

Table 2. Analyses of Pill-count Adherence at Different Cut-off Points

| **Cut-off point** | **Control group** | | **Intervention group** | | χ^2^ | P **value** |
| --- | --- | --- | --- | --- | --- | --- |
|  | **N'=77** | | **N’=89** | |  |  |
|  | **n** | **%** | **n** | **%** |  |  |
| ＜0.7 | 41 | 48.8 | 43 | 51.2 | 0.402 | 0.526 |
| ≥0.7 | 36 | 43.9 | 46 | 54.8 |  |  |
|  |  |  |  |  |  |  |
| ＜0.8 | 43 | 48.9 | 45 | 51.1 | 0.462 | 0.497 |
| ≥0.8 | 34 | 43.6 | 44 | 56.4 |  |  |
|  |  |  |  |  |  |  |
| ＜0.9 | 46 | 48.4 | 49 | 51.6 | 0.373 | 0.543 |
| ≥0.9 | 31 | 43.7 | 40 | 56.3 |  |  |
